# Root Canal Anatomy and Morphology of Mandibular First Molars in a Selected Iranian Population: An *In Vitro* Study

**DOI:** 10.22037/iej.2017.18

**Published:** 2017

**Authors:** Nahid Mohammadzadeh Akhlaghi, Zohreh Khalilak, Mehdi Vatanpour, Saman Mohammadi, Sakineh Pirmoradi, Mahta Fazlyab, Kamran Safavi

**Affiliations:** a *Department of Endodontic, Dental Branch, Islamic Azad University, Tehran, Iran; *; b *Private Practice, Tehran, Iran; *; c *Iranian Center for Endodontic Research, Research Institute of Dental Sciences, Dental School, Shahid Beheshti University of Medical Sciences, Tehran, Iran; *; d *Department of Endodontic, University of Connecticut, Connecticut, USA*

**Keywords:** Iranian Population, Mandibular First Molar, Root Canal Anatomy, Root Canal Morphology, Tooth Clearing

## Abstract

**Introduction:**

The aim of this study was to evaluate root canal anatomy of mandibular first molars (MFM) in a selected Iranian Population using clearing technique.

**Methods and Materials:**

A total of 150 extracted MFMs were cleared. The root canal morphology (including the root numbers and root length) and the anatomy of the root canal system (including is the number and type of canals based on Vertucci’s classification, canal curvature according to Schneider's method and the presence of isthmus) was evaluated using the buccolingual and mesiodistal parallel x-rays and stereomicroscope. The data were analyzed using the chi-square test.

**Results:**

Two and three roots were present in 96.7% and 33% of the teeth, respectively (*P*=0.0001). All the teeth (100%) had two canals in the mesial root, while 61.3% of the samples had one distal root canal (*P*=0.006). The root canal configuration in the mesial canal included type IV (55.3%) and type II (41.3%) (*P*=0.0001). In doubled-canalled distal roots, 68.8% and 24.3% were type II and type IV, respectively (*P*=0.0001). Isthmii were observed in 44.6% of mesial and 27.3% of distal roots (*P*=0.0001).

**Conclusion:**

The notable prevalence of type IV configuration in both roots of mandibular first molars, presence of isthmus and root curvature, necessitates the careful negotiation and cleaning of all accessible canal spaces.

## Introduction

Successful outcome of endodontic treatment largely depends on proper cleaning and shaping of the entire root canal system. Thorough understanding of the root canal morphology and configuration is mandatory [1]. Mandibular first molars are amongst the most commonly teeth requiring endodontic treatment due to their early emergence in the oral cavity and subsequent caries [2]. Like any other tooth, a clear and thorough knowledge of the root canal morphology and anatomy of mandibular first molars, will help the clinician with conducting a proper and standard treatment through enabling to foresee possible variations that potentially challenge the treatment outcome [3-6].

Mandibular first molars commonly have two roots and three root canals [2, 7]. However, due to genetic, ethnic and gender varieties, a wide range of anatomic and morphological variations can be encountered [7]. Commonly used methods to evaluate root canal morphology include root canal staining and tooth clearing [8-11], plastic injection [12], conventional and digital radiography [13, 14] and radiopaque gel infusion and radiography [15, 16]. Recently, cone-beam computed tomography (CBCT) and micro-CT images have been found to be useful in providing accurate three-dimensional anatomic details [17-19].

Numerous reports on root canal morphology of mandibular molars were from different countries and variable ethnicities [5-7, 10, 20-22]. These studies have evaluated the root and canal morphology of the mandibular first molars in Korean [23, 24], Indian [25, 26], Chinese [27], German [28], Jordanian [29] and Sudanese [30] populations. 

Iranian national surveys in this regard are not numerous. Baziar *et al. *[31], reported the management of a six-canaled mandibular first molar with three canals in the distal root using CBCT. In a case series, Aminsobhani *et al. *[32], reported the endodontic treatment of 27 mandibular first and second molars with the third mesial canal (middle mesial canal) that in all cases joined to mesiobuccal or mesiolingual canals. Ghoddusi *et al. *[33], reported a rare case of a mandibular first molar with six canals (two mesial and four distal canals in two distal roots). In the one and only study in this regard, Razmi *et al. *[2] evaluated the number of distal roots and canals in 310 mandibular first molars and their internal anatomy, using radiographies. They reported a 4.5% incidence of two distal roots (100% Vertucci’s type I). According to Vertucci's classification 54.9% of the teeth with one distal root type I, 19% type II, 1.9% type III, 14.2% type IV, 4.2% type V, 1% type VI, 0.3% type VII and 0% type VIII [2]. However, the latter study just focused on the distal root of mandibular first molars. Shahi *et al. *[34] conducted a study on the anatomy and root morphology of mandibular first molars in a Iranian population using clearing and staining. They showed that 98.56% and 1.44% of the teeth were two-rooted and three-rooted, respectively. Also 65.56% of mandibular first molars had three canals, while 31.57% had four and 2.87% had two canals. Mesial roots with two canals were of type II (41.87%), type III (0.49%), type IV (53.69%) and type V (3.94%). Distal roots were type I (68.42%), type II (11.96%), type III (1.99%), type IV (17.22%) or type V (0.48%) based on Vertucci’s classification. 

Due to the lack of a complete national morphological survey in this regard and the different results of the latter two national studies, the aim of this *in vitro* cross-sectional study was to evaluate the anatomy and morphology of mandibular first molars (including the number of roots and root canals, canal curvature and presence of isthmuses) in an Iranian population using clearing technique.

## Materials and Methods

This study was conducted on a total number of 150 extracted mandibular first molars with mature apices, free of any crack, internal/external resorption and without root filling material. The teeth were collected from dental clinics and a selected Iranian population of variable age and gender during 2013 and 2014. The teeth were scrubbed under tap water for 30 min to remove adherent soft tissues and were then disinfected in 5.25% NaOCl for 1 h. The teeth were stored in sterile normal saline until the assessment time. 

The teeth were coded and a data form was assigned to each sample. The number of the roots and root lengths (measured in millimeters from anatomic apex to cemento enamel junction) were assessed and recorded. 

Then for each sample, access cavity preparation was done and the pulp chamber space was rinsed with 5.25% NaOCl solution for removal of the necrotic tissues. After locating the canal orifices and canal negotiation using a #10 K-file (Dentsply Maillefer, Ballaigues, Switzerland), two parallel digital radiographies (Digora, Soredex, Orion Corporation Ltd., Helsinki, Finland) were taken in buccolingual and mesiodistal directions with a #15 K-file placed to the working length. The images were assessed in Autocad software (2015, Autodesk, San Rafael, Calif, USA) and determination of root canal curvature was done according to Schneider’s method [35]. 

The root canals were subsequently irrigated with 5.25% NaOCl solution and distilled water using a 30-gauge needle. After allowing the samples to dry for 24 h, the same syringe and needle was used for injection of Indian ink (Pelikan, Tehran, Iran). Then for complete distribution of the ink in entire root canal the teeth were stored in a vertical position by placing them in the head of high vacuum saliva ejector. Distribution of ink in the apical foramina indicated the end of the process.

Decalcification process was performed by immersion of teeth in 5% nitric acid for 3 days at room temperature. The solution was refreshed daily and the teeth were washed under running tap water for 10 min. The teeth were dehydrated by immersing subsequently in 80, 90 and 100% ethanol for 1 day. After drying with tissue paper, the samples were inserted in 50% methyl salicylate for 5 h to make them transparent.

The transparent samples were evaluated using a stereomicroscope (Nikon SMZ1500, Nikon Corporation, Tokyo, Japan) under 10× magnification. The number of root canals, the type of canals based on Vertucci’s classification and the isthmii at the distances of 2, 4 and 6 mm from the apex, were recorded for each tooth [1]. A single operator did all the procedure and microscopic evaluation was double-checked by a second observer. The data were analyzed by the chi-square test using SPSS software (Statistical Package for Social Science, SPSS, version 18.0, SPSS, Chicago, IL, USA). The level of significance was set at 0.05. 

**Table 1:** Distribution of the root canal number and configuration in 150 mandibular first molars

	**Number of canals (%)**			**Root canal configuration (Vertucci’s classification) (%)**							
**Root**	**1**	**2**	**3**	**Type I**	**Type II**	**Type III**	**Type IV**	**Type V**	**Type VI**	**Type VII**	**Type VIII**
**Mesial**	0	100	0	0	41.3	3.3	55.3	0.7	0	0	0
**Distal**	61.3	38.7	0	61.3	26.6	2	9.4	0.65	0	0	0

## Results

A total of 150 samples were ready for final evaluation. Among these, 145 teeth (96.7%) had two roots while 5 (3.3%) were three-rooted with a separate distolingual root (*P*=0.0001). In all double-rooted teeth the mesial root had two canals while the distal root had one canal in 61.3% and two canals in 38.7% of the samples (*P*=0.006). In three-rooted samples, the distobuccal and distolingual root had a single canal.

In mesial roots, the most frequent canal types were type IV (55.3%), type II (41.3%), type III (3.3%) and type V (0.7%) (P=0.0001). In distal roots with two canals, the frequent anatomies were Vertucci’s type II (68.8%), type IV (24.3%), type III (5.18%) and type V (1.72%) (*P*=0.0001) (Table 1).

Isthmii were found in 44.6% of mesial roots and 27.3% of distal roots (*P*=0.0001). In 2, 4 and 6 mm levels from the apex of mesial roots, isthmii were found in 22.5%, 41.7% and 35.8% of the samples, respectively. In distal roots, isthmii were found in 34.3%, 36.5% and 29.2% of teeth in 2-, 4- and 6-mm distances from the apex, respectively.

The mean root canals curvatures for each of the canals and the mean root lengths are presented in Tables 2 and 3, respectively.

## Discussion

This cross-sectional *in vitro* study evaluated the root canal anatomy and morphology of extracted mandibular first molars from a selected Iranian population. In this study the clearing technique was used as suggested by Singh and Pawar [8], Okumara and Tsurukichi [36] and Robertson *et al*. [37], to determine the root canal morphology, anatomy and presence of isthmii. Moreover, root lengths and curvatures were also determined by radiographic method. Apart from being inexpensive and easy to conduct, other important advantages of clearing technique include retaining the original form of the canal, enabling the assessment of canal form and isthmus and maintenance of the samples for long time [38].

Kim *et al. *[23], conducted a retrospective study on root and canal morphology of the mandibular first molars in a Korean population by analysis of a large number of CBCT images. Among the examined molars, 25.82, 73.51 and 0.67% had 3, 2 roots, and 1 roots, respectively.

**Table 2:** The mean (SD) of canal curvature in mandibular first molars based on Schneider’s method

	**Canal curvature (degrees)**	
**Canal**	**Buccolingual**	**Mesiodistal**
**Mesiobuccal**	13.8 (6.78)	11.8 (5.4)
**Mesiolingual**	9 (5.1)	7.53 (4.39)
**Distobuccal**	5 (4.01)	5.3 (3.6)
**Distolingual**	7.16 (4.95)	2.7 (1.19)
**Distal**	21.5 (5.3)	18.5 (8.4)

In the mesial and distal roots, type IV and type I canal was the most frequent morphology. They reported a high prevalence of four-canaled molars with separate distolingual (DL) root and/or separate DL canals [23]. In another study on Korean population using CBCT, Kim and Yang [24] evaluated the incidence of separate DL root and a separate DL canal in the distal root. They reported a higher prevalence of two distal canals and two distal roots on the right side compared to the left side (26.6% and 19.0%, respectively). This evaluation showed significantly lower prevalence of one distal root with two distal canals [24]. Chourasia *et al. *[25], determined the number of roots, root canals, root canal configurations and frequency of isthmii and apical deltas in mandibular first molars in an Indian population, using clearing technique *in vitro*. They reported two mesial and distal roots and a separate DL root in 94.6 and 5.3% of the mandibular first molars. Moreover, 36% of the two-rooted molars had separate DL canals [25]. Garg *et al. *[26] also reported the prevalence of three-rooted mandibular first molars to be 5.97% in Indian population (6.88% for female and 4.89% for male patients). Ahmed *et al. *[30] stated that 59% of the mandibular first molars in Sudanese population had four canals with 3% having a third distolingual root. Also the most common canal configurations were Vertucci’s type IV (73%) and type II (14%) and the prevalence of isthmii was reported to be 65%. Al-Qudah and Awawdeh [29] conducted a similar study on Jordanian population and reported the majority of mandibular first molars to have three (48%) or four (46%) canals, whilst 4% had a third distolingual root. The most common configuration in the mesial root was type IV (53%) and in distal root was type I (54%). Schäfer *et al. *[28] stated that the overall incidence of three-rooted mandibular first molars in a selected German population was rare (1.35%). Moreover, all three-rooted molars occurred unilaterally (0.80% in left and 0.57% in right side). Zhang *et al. *[27] reported the majority of mandibular first molars (70%) had two separate roots and three roots were identified in 29% of first molars. Three canals were found in 56% of teeth and most distal roots had a simple type I configuration. 

The present study showed that the majority of mandibular first molars of the Iranian population had two roots (96.7%) whereas three-rooted cases were less common (3.3%). All the mesial roots had two root canals and based on Vertucci’s classification, type II and IV were the most common types.

**Table 3:** The mean (SD) of root length in mandibular first molars

**Root**	**Root length (mm)**
**Mesial**	15.68 (2.88)
**Distal**	15.1 (3.5)
**Distolingual**	8 (1.6)

Distal roots presented more variable canal types. The most common type was type I (61.3%) and the reset (38.7%) consisted of less frequent types including types II, III, IV and V.

There are variable methods for assessing the morphology of teeth and root canal system. Some studies showed the superiority of clearing technique over radiographic techniques which have limited value in studying the anatomy of the root canal system [39, 40]. Lu *et al*. [41] used cross-sectioning method which is complicated. A study by Neelakantan* et al*. [42] concluded that CBCT was as accurate as *in vitro* techniques such as canal staining and clearing technique, which necessitate tooth extraction. However, in a recent study by Lee *et al*. [43] it was stated that in micro-CT, the combination of two-dimensional projection with minimum intensity and three-dimensional volume rendering for reconstruction images, provide a more detailed canal morphology than the clearing technique. This state may be true, but the expenses and high beam dosage of tomography are not cost effective.

## Conclusion

Based on the results of this study, mandibular first molars have complicated root canal system with widely found isthmii and anatomical variations, and therefore require more attention in access cavity preparation for negotiating all root canal orifices, and during cleaning, shaping and obturation.
